# Ultrasound-Guided Hyaluronidase Injections for the Management of Filler-Induced Arterial Ischemia: A Pictorial Case Series and Systematic Review of Literature

**DOI:** 10.1093/asjof/ojaf125

**Published:** 2025-11-04

**Authors:** Narges Azizi, Nasim Tootoonchi, Faezeh Khorasanizadeh, Maryam Nasimi, Amir Hooshang Ehsani, Mahshid Sadat Ansari, Narges Ghandi, Shahin Hamzelou, Ximena Wortsman

## Abstract

The use of fillers for cosmetic purposes has increased in recent years. Although generally considered safe, fillers are not exempt from complications, including ischemic events. Arterial ischemia is a rare but potentially serious complication that requires prompt recognition and management. Ultrasound-guided hyaluronidase injection is emerging as a precise and effective treatment approach. The authors present a pictorial case series of filler-induced arterial ischemia managed with ultrasound-guided hyaluronidase injections, and systematically review the literature on ultrasound findings, enzyme dosage, time to resolution, and overall effectiveness of this approach. They present 3 cases of filler-induced arterial ischemia in the frontal, nasal, and temporal regions with visible intravascular thrombosis managed with ultrasound-guided intravascular hyaluronidase injections. Additionally, the authors systematically reviewed literature from PubMed/Medline, Scopus, Embase, and Web of Science up to August 2024 to evaluate the effectiveness, dosage, and ultrasound techniques utilized in the ultrasound-guided treatment of vascular complications from hyaluronic acid fillers. Following Preferred Reporting Items for Systematic Reviews and Meta-Analyses guidelines, we screened 88 studies, finally including 9 studies after full-text evaluation. Data from these studies, alongside our 3 cases (totaling 83 cases), showed rapid resolution of symptoms and ultrasound abnormalities following ultrasound-guided hyaluronidase injections. Delayed hyaluronidase administration was associated with slower or incomplete recovery, highlighting the importance of early intervention. Ultrasound-guided hyaluronidase injections effectively resolve arterial ischemia caused by filler injections, with early intervention significantly enhancing outcomes. Prompt diagnosis and timely ultrasound-guided intervention should be emphasized in clinical practice. Further large-scale randomized studies are necessary to establish standardized treatment protocols for dosage and timing.

**Level of Evidence:** 3 (Therapeutic) 
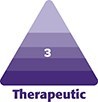

The use of fillers for cosmetic purposes has increased in recent years. Although generally safe, fillers are not exempt of complications, including ischemic events. Arterial ischemia is a rare but serious complication that requires prompt recognition and management. Vascular complications from filler injections can arise through 3 main mechanisms: (1) direct injection of the filler into an artery, (2) vessel compression because of excess filler, and (3) arterial spasm.^1-3^ Although the exact mechanism of vascular spasm remains unknown, it might result from reduced perfusion pressure, hypoxia, or reactive substances released during ischemia.^3^ These complications can lead to severe outcomes such as permanent tissue damage or blindness, especially in high-risk areas like the glabella, nose, nasolabial folds, and forehead.^3^

Ultrasound is increasingly utilized in dermatology to map patient-specific vascular anatomy and identify common cosmetic fillers, significantly reducing the risk of intravascular injection when performed before filler placement.^4,5^ Ultrasound also serves as a critical diagnostic tool for assessing filler-related vascular complications, providing clear visualization of filler deposits and blood flow impairment.^3,6,7^

In this study, we aim to illustrate 3 pictorial series of filler-induced arterial thrombosis, along with detailed images of the ultrasound-guided hyaluronidase injection process. Additionally, we conducted a systematic review to evaluate the effectiveness of ultrasound-guided hyaluronidase injections in managing filler-induced vascular impairment.

The authors confirm that this case series follows the Declaration of Helsinki. All patients gave written informed consent to share their case details. Ethical approval was not needed for this type of retrospective case series. The patients gave written informed consent for the publication of their clinical images.

## CASE PRESENTATION

### Case 1

**Figure 1.**
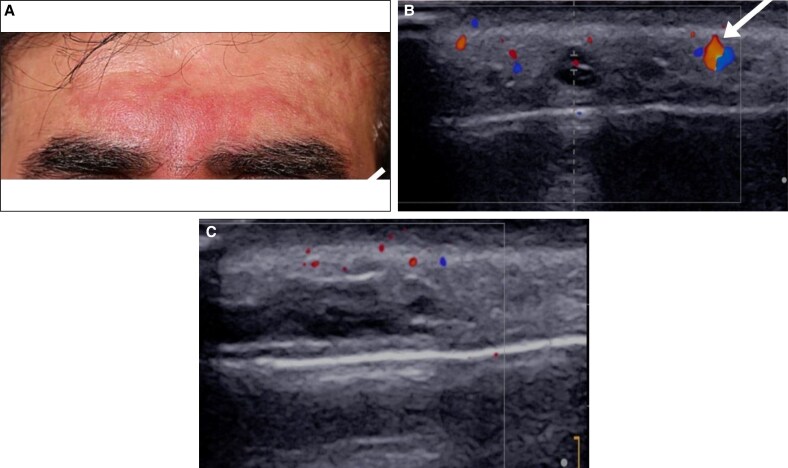
Baseline clinical presentation and ultrasound findings (Case 1, a 45-year-old man). (A) Clinical photograph showing erythema and pallor in the glabellar region. (B) Transverse color Doppler ultrasound showing avascularity in the right supratrochlear artery compared with normal flow on the contralateral side (arrow). (C) Longitudinal color Doppler ultrasound view.

A 45-year-old man presented 1 day following a glabellar hyaluronic acid filler injection for cosmetic wrinkle correction. He reported moderate pain and pruritus in his forehead. Clinical examination revealed erythema and tenderness localized in the glabella region. Color Doppler ultrasound using a 20 MHz linear probe (Supersonic Aixplorer MAC20, Hologic, Marlborough, MA) revealed a noncompressible tubular structure above the right medial eyebrow without detectable blood flow, consistent with acute thrombosis of the right supratrochlear artery, potentially compressing the adjacent vein (Figure 1).

**Figure 2.**
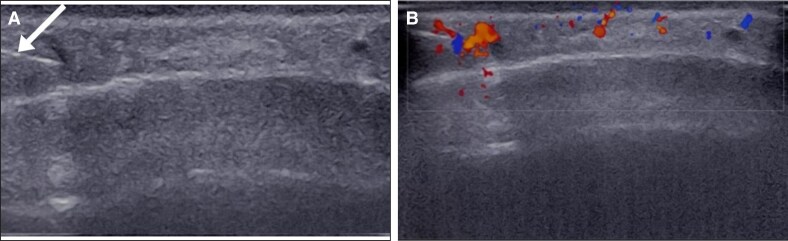
Ultrasound-guided hyaluronidase injection (Case 1, a 45-year-old man). (A) Transverse ultrasound view illustrating the guidance of hyaluronidase injection with a 2.5 cc syringe needle (arrow). (B) Immediate restoration of arterial flow during the hyaluronidase injection.

To address this, hyaluronidase (300 units from a Hyalase vial of 1500 international units [IU]) was injected under ultrasound guidance using a 25-G, 25 mm needle attached to a 2 cc syringe. This approach was necessary because of the deep location of the artery beneath the epidermal surface. During real-time ultrasound-guided injection rebound flow was detected in the right side, immediately (Figure 2). Follow-up ultrasound was performed at 1 h and 24 h after the initial hyaluronidase injection. Because no clinical or sonographic evidence of ischemia was present at those time points, no further injections were indicated.

**Figure 3.**
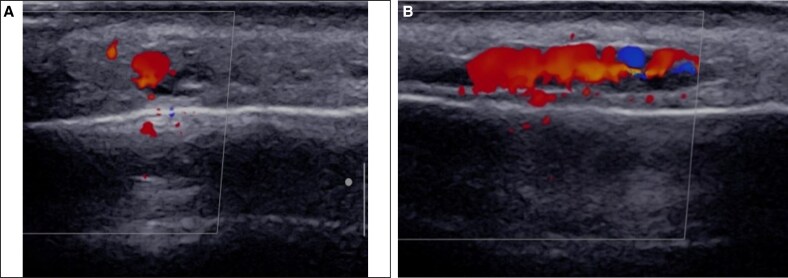
Clinical and ultrasound evaluation 1 week after hyaluronidase treatment (Case 1, a 45-year-old man). (A) Resolution of clinical symptoms. (B) Transverse color Doppler ultrasound showing restored flow within the supratrochlear artery; a small anechoic defect adjacent to the artery, consistent with residual hyaluronic acid, is noted.

A follow-up ultrasound 1 week later showed complete compressibility of the right supratrochlear artery and vein with fully restored arterial and venous blood flow (Figure 3). Spectral Doppler analysis revealed a low resistance index, indicative of compensatory hyperemia and improved perfusion. Ultrasound also identified a residual anechoic area surrounding the vessels, suggestive of small hyaluronic acid remnants. Consequently, another ultrasound-guided hyaluronidase injection of 300 units was administered, resulting in complete resolution of these residual deposits. Throughout a 6-month follow-up period, the patient experienced no recurrence of symptoms.

### Case 2

**Figure 4.**
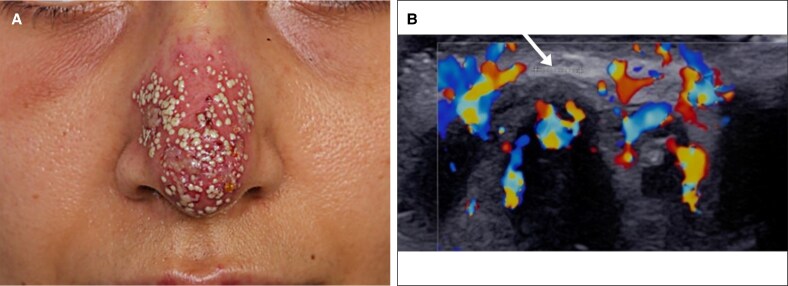
Baseline clinical presentation and color Doppler ultrasound images (Case 2, a 32-year-old woman). (A) Clinical image displaying erythematous-violaceous plaques and pustulosis in the nose. (B) Transverse color Doppler ultrasound image showing a noncompressible, tubular structure in dorsal nasal artery (arrow).

A 32-year-old woman presented 2 days after nasal hyaluronic acid filler injection (EPTQ, monophasic cross-linked hyaluronic acid filler; JETEMA Co., Ltd, Seoul, South Korea), exhibiting severe tenderness, erythematous-violaceous plaques with a reticular pattern, pustulosis, and erosions. Color Doppler ultrasound (20 MHz linear probe, Supersonic Aixplorer MAC20) showed a noncompressible tubular structure on the dorsum of the nose. No blood flow was detected, suggesting acute thrombosis of the dorsal nasal artery. Additionally, soft tissue hypovascularity was evident compared with the normal left side (Figure 4).

**Figure 5.**
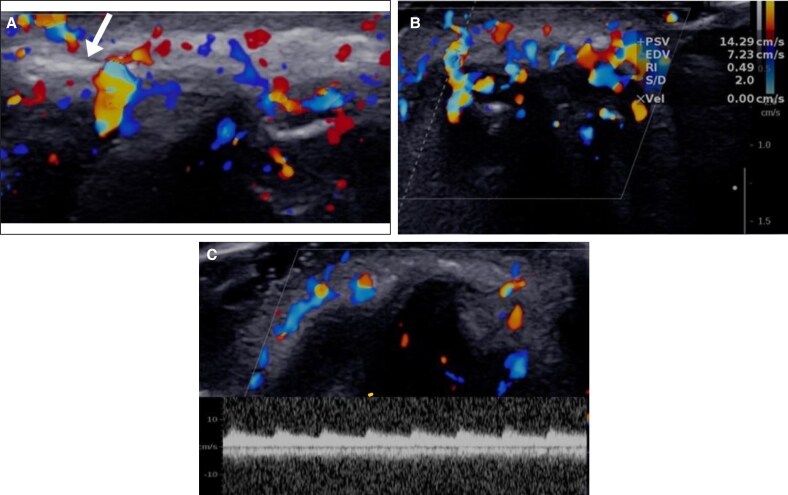
Ultrasound-guided hyaluronidase injection (Case 2, a 32-year-old woman). (A) Transverse ultrasound view illustrating the guidance of hyaluronidase injection (arrow) with a 2.5 cc syringe needle. (B,C) Immediate restoration of arterial flow during the hyaluronidase injection. The spectral analysis (B) displaying elevated higher Peak systolic velocity (PSV = 14cm/s) and resistance index (RI = 0.49) compared with normal side (C) (PSV = 8cm/s and RI = 0.3).

Under ultrasound guidance, 150 units of hyaluronidase were injected using a 2 cc syringe with a BD insulin needle, 31-G, 12.7 mm length, resulting in an immediate increase in soft tissue vascularity; however, no flow was initially detected in the thrombosed artery. A second injection of 150 units of hyaluronidase was administered 15 min later, after which rebound flow in the dorsal nasal artery was observed, showing increased peak systolic velocity and a resistance index comparable to the normal contralateral side. Clinically, the violaceous discoloration faded to pink, and the patient reported a reduction in nasal pain, tenderness, and congestion (Figure 5). Additionally, the patient's pustules were drained to minimize scar formation.

**Figure 6.**
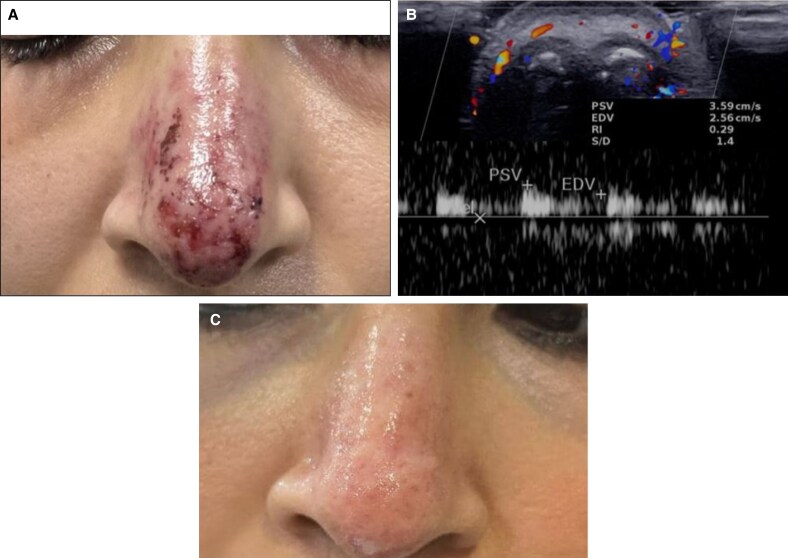
Clinical and ultrasound follow-up 2 days and 1 week after hyaluronidase treatment (Case 2, a 32-year-old woman). (A) Resolution of clinical symptoms 2 days posttreatment. (B) Transverse color Doppler ultrasound showing restored flow. (C) Clinical image at 1-week follow-up.

A follow-up 2 days posttreatment showed a significant reduction of erythema and erosions, complete resolution of pain, and normal blood flow with peak systolic velocity and resistance index comparable to the normal side. Complete clinical resolution was evident after 1 week (Figure 6).

### Case 3

A 60-year-old woman with a 30-year history of diabetes presented 10 days following bilateral temporal hyaluronic acid filler injections. Initially, she experienced a temporofrontal headache, which she ignored. Physical examination revealed erythema and ecchymosis in the left temporal area, along with superficial erosion and crusting. She was initiated on aspirin, cephalexin, and mupirocin.

**Figure 7.**
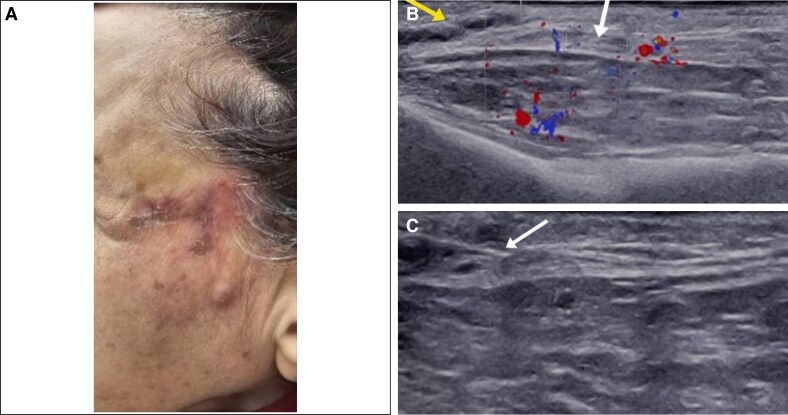
Baseline clinical and ultrasound evaluation (Case 3, a 60-year-old woman with diabetes), (A) Clinical image displaying erythematous-violaceous plaques and pustulosis in the temporal region. (B) Transverse color Doppler ultrasound showing a noncompressible, tubular structure at the distal end of superficial temporal artery (white arrow) and adjacent hyaluronic acid deposits (yellow arrow). (C) Transverse ultrasound view illustrating the guidance of hyaluronidase injection with a 2.5 cc syringe needle (arrow).

Color Doppler ultrasound, performed with a 20 MHz linear probe (Supersonic Aixplorer MAC20), showed a noncompressible, dilated tubular structure within the left superficial musculoaponeurotic system. The absence of blood flow on color Doppler suggested acute thrombosis of the left superficial temporal artery. Multiple hypoechoic pseudocystic structures, indicative of hyaluronic acid filler accumulation, were also observed adjacent to the artery (Figure 7).

**Figure 8.**
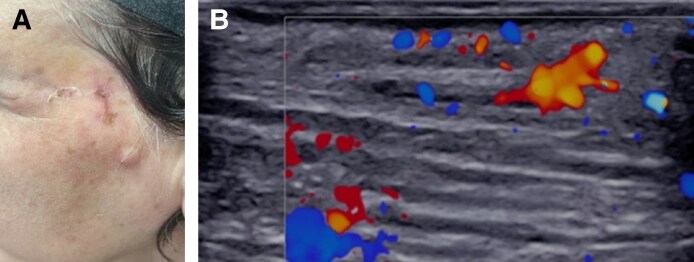
Clinical and ultrasound evaluation 4 days after initial hyaluronidase treatment (Case 3, a 60-year-old woman with diabetes). (A) Resolution of the patient's clinical symptoms. (B) Transverse color Doppler ultrasound showing partial flow restoration in superficial temporal artery. (C) Clinical image of patient 2 days after injection of additional 300 units hyaluronidase.

Under ultrasound guidance, 450 units of hyaluronidase were injected twice using a 2 cc syringe with an insulin needle, 30-G, 8 mm length, to restore blood flow in the superficial temporal artery. Following these injections, the patient's pain subsided and the erythema diminished. Four days later, sonographic examination revealed persistent thrombosis in the left superficial temporal artery, with minimal blood flow and a reduced volume of hyaluronic acid filler around the vessel. However, the patient reported no pain, and the ulcers were healing. An additional 300 units of hyaluronidase were injected, resulting improvement in blood flow. It was concluded that the thrombosis was likely a bland superficial thrombophlebitis. The delayed response to hyaluronidase was attributed to the chronicity of the symptoms, given the prolonged interval between filler injection and hyaluronidase administration. Nevertheless, the patient's symptoms completely resolved within 1 week (Figure 8).

**Figure 9.**
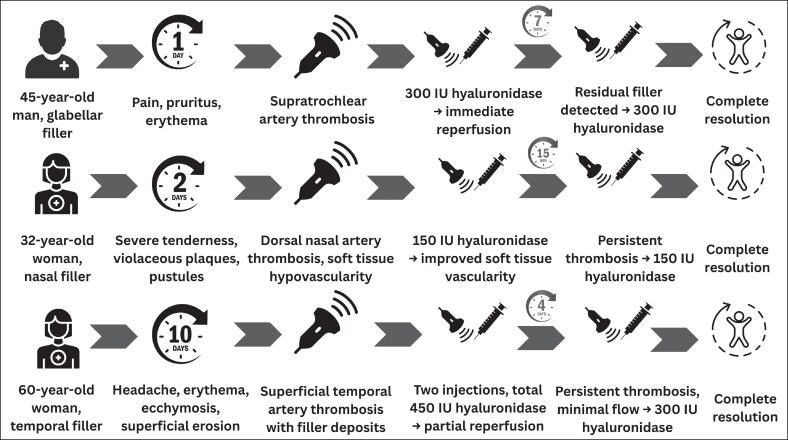
Treatment timelines.

The treatment timeline for each case is summarized in Figure 9. Symptom severity was retrospectively quantified using simple ordinal scales. Pain intensity was rated on a 0 to 3 scale (0 = none, 1 = mild, 2 = moderate, 3 = severe), and tissue damage was graded on a 0 to 3 scale (0 = none, 1 = superficial changes such as pustules or erosions, 2 = partial-thickness necrosis, 3 = full-thickness necrosis). All patients signed an informed consent for the publication of the data and figures.

### Review of Literature

We conducted a systematic literature review following the Preferred Reporting Items for Systematic Reviews and Meta-Analyses (PRISMA) guidelines, registered in PROSPERO (CRD42024583473).^8,9^ We searched the Embase, PubMed/Medline, Scopus, and Web of Science databases for studies available up to August 2024 using the following MeSH terms: “Dermal Fillers,” “Fillers,” “Hyaluronic Acid,” “Ischemia,” “Necrosis,” “Hyaluronoglucosaminidase,” “Ultrasonography,” “Ultrasound,” and “Treatment Outcome” (Supplemental Tables 1-3).

Original English-language studies were included if they reported ultrasound-guided hyaluronidase treatment outcomes for vascular complications following hyaluronic acid filler injections. Reviews, editorials, nonoriginal studies, studies focusing on nonvascular complications, studies involving fillers other than hyaluronic acid, and those not employing ultrasound-guided hyaluronidase or lacking relevant outcomes were excluded. Two independent reviewers screened articles, resolved disagreements through consultation with a third reviewer, and performed data extraction using a template. Extracted data included demographics, publication details, injection sites, hyaluronidase dosage, clinical presentations, outcomes, and follow-up durations.

## RESULTS

**Figure 10.**
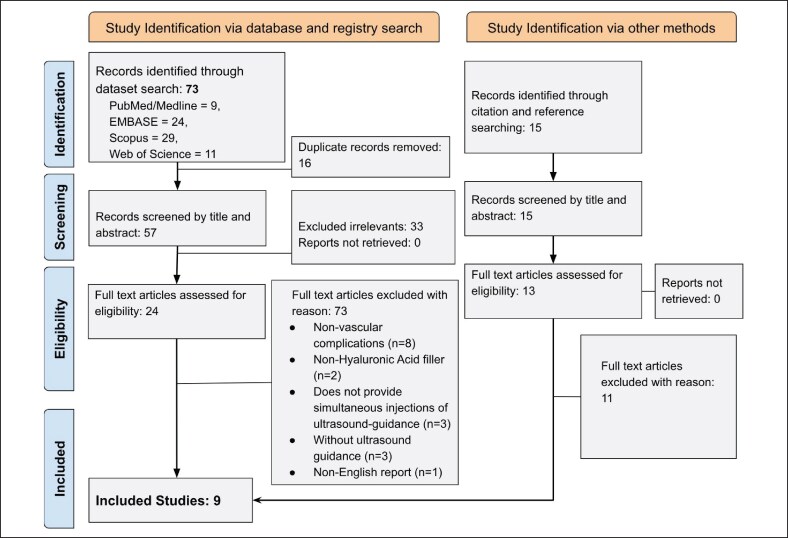
Preferred Reporting Items for Systematic Reviews and Meta-Analyses 2020 flow diagram.

The study selection process, detailed in Figure 10, followed the PRISMA guidelines.^10^ Our initial search of multiple databases along with manual tracking, identified 88 papers. After removing duplicates, 72 papers remained for screening. Based on the review of titles and abstracts, 35 papers were excluded for not meeting the study criteria. A full-text review on the remaining 37 studies resulted in the inclusion of 9 studies. The characteristics of these included studies are summarized in Supplemental Table 4.

Together with our 3 cases, we evaluated a total of 83 cases. All patients showed complete recovery except for 1 patient who experienced a delay of 38 days, another with a delay of 56 days, and 2 patients with unclear outcomes. Similarly, among our presented cases, particularly in Case 3, delayed responses were observed when hyaluronidase injection occurred after prolonged intervals between filler injections and treatment initiation. Apart from these exceptions, symptoms were resolved almost immediately within 15 min in other cases. Hyaluronidase doses administered varied, with 67 patients receiving doses ranging from 30 to 150 IU, 2 patients receiving 300 IU, ten patients administered doses between 300 and 750 IU, 1 patient receiving 900 IU, and another patient receiving 1500 IU. Outcomes for 2 patients were unclear. A detailed case-by-case summary from the reviewed literature is provided in Supplemental Table 5.

## DISCUSSION

In this study, data from 80 patients across 9 studies and 3 cases from our center show the efficacy of ultrasound-guided hyaluronidase injections, achieving rapid symptom resolution with relatively low enzyme doses in managing filler-induced arterial ischemia. Detailed ultrasound imaging in our 3 cases revealed features of filler-induced ischemia, including intravascular thrombosis, loss of vessel compressibility, and the absence of Doppler flow signals at baseline. Subsequent real-time ultrasound-guided hyaluronidase injections resulted in immediate restoration of arterial flow, clinical improvement within days, and sustained resolution over follow-up periods. Our results emphasize that timely ultrasound-guided hyaluronidase administration not only enhances clinical outcomes but also effectively reduces the required dosage. In contrast, delayed intervention negatively impacts treatment efficacy and prolongs recovery.

Preventing filler complications requires a comprehensive understanding of facial anatomy, using appropriate injection techniques, like using cannulas instead of needles in high-risk areas, and prompt action when vascular complications occur.^11^ Current guidelines recommend immediate administration of high-dose hyaluronidase (500-4500 IU), a “flooding” technique intended to thoroughly saturate ischemic tissues and facilitate enzyme penetration into occluded vessels.^12-15^ However, this method has limitations, including dependence on the injector's visual assessment and clinical expertise, and increased risk of adverse reactions like urticaria and angioedema, because of the enzymatic degradation of glycosaminoglycans by hyaluronidase.^2,16^

Ultrasound-guided injections, by contrast, enable precise delivery of lower hyaluronidase doses directly to affected arteries, rapidly restoring blood flow, minimizing adverse effects, and preserving aesthetic outcomes. Furthermore, ultrasound provides immediate feedback on the success of the treatment, enabling real-time adjustments if necessary. This might reduce the risk of further ischemia because of partially degraded filler moving downstream, a significant concern with the flooding method.^17^

Schelke et al first proposed an alternative ultrasound-guided treatment protocol for managing adverse vascular events following facial filler injections.^6^ In a subsequent study, they evaluated the effectiveness of an ultrasound-guided compared with flooding protocol involving 39 patients.^2^ The targeted ultrasound-guided approach utilized only 123 IU of hyaluronidase, significantly less than the average 1519.4 IU required by the flooding method, and resulted in a more rapid resolution of patient symptoms.^2^

Integrating ultrasound into cosmetic dermatology requires anatomical and sonographic expertise and multidisciplinary collaboration. At our academic referral center, a significant challenge is managing patients previously treated elsewhere with high-dose, blind hyaluronidase injections (flooding protocol), which can paradoxically result in decreased rather than increased blood flow on color Doppler ultrasound because of previous interventions. Thus, early referral without pretreatment is critical for optimal outcomes. For patients without previous treatments, we perform urgent color Doppler ultrasound conducted by an expert dermatologic radiologist. If clinical symptoms or ultrasound findings indicate soft tissue ischemia, we promptly initiate ultrasound-guided hyaluronidase injections, typically using a dose of 350 units, by dermatologist and radiologist under real-time ultrasound monitoring. If blood flow is not restored after the initial injection, additional injections are repeated as necessary.

Currently, there is a shortage of prospective studies addressing the optimal dose and timing for ultrasound-guided hyaluronidase treatment. Detailed procedural descriptions and comprehensive ultrasound imaging that document immediate therapeutic effects remain limited in the existing literature. In this study, we specifically address these gaps by providing detailed illustrations from 3 cases, depicting intravascular thrombosis, the process of ultrasound-guided intra-arterial hyaluronidase injections, and sequential color Doppler sonographic features observed before, during, and after injections.

Although limited patient numbers in studies focusing on filler-induced ischemia restrict definitive conclusions, current evidence highlights the need for timely referral, avoidance of previous interventions, and management by a multidisciplinary team. The heterogeneity across studies in anatomical treatment sites, ultrasound methods, and follow-up duration underscores the necessity for standardized treatment protocols. Large-scale randomized controlled trials are needed to establish definitive guidelines regarding dosage, timing, and frequency of ultrasound-guided hyaluronidase injections.

## CONCLUSIONS

In this study, the authors highlight the importance of prompt recognition and management of filler-induced ischemic events. Ultrasound-guided hyaluronidase injection is an effective treatment option for arterial ischemia after filler injection. Early recognition, established guidelines, and multidisciplinary collaboration enhance clinical outcomes. Healthcare professionals should recognize the effectiveness of timely ultrasound-guided hyaluronidase injections in resolving vascular complications.

## Supplementary Material

ojaf125_Supplementary_Data
