# Supplementary material for: Ultrasound-Guided Hyaluronidase Injections for the Management of Filler-Induced Arterial Ischemia: A Pictorial Case Series and Systematic Review of Literature
Source: Aesthet Surg J Open Forum. 2025 Nov 4;7:ojaf125. doi: 10.1093/asjof/ojaf125 (PMC12586333; doi:10.1093/asjof/ojaf125)
Supplement: ojaf125_Supplementary_Data [file ojaf125_supplementary_data.docx]

This is the supplementary information of “***Ultrasound-Guided Hyaluronidase Injections for Management of Filler-Induced Arterial Ischemia: A Pictorial Case Series and Systematic Review of Literature*** ” by Nasim Tootoonchi, Narges Azizi, Maryam Nasimi, Faezeh Khorasanizadeh, and Ximena Wortsman.

*Table of content*

**Table S1:** MeSH terms

**Table S2:** tiab terms

**Table S3:** Search strategy used for Pubmed/Medline, Scopus, Embase, and Web of Science (Date: 8.24.2024).

**Table S4:** Characteristics of the included studies

**Table S5:** Case by case presentation from the included studies

**Table S1:** MeSH terms

| #1 Population: Patients presenting with vascular complications due to hyaluronic acid dermal filler injections | “Dermal Fillers” [MeSH] OR “Hyaluronic Acid” [MeSH] OR “Ischemia” [MeSH Terms] OR “Necrosis” [MeSH] |
| --- | --- |
| #2 Intervention: Administration of hyaluronidase | “Hyaluronoglucosaminidase” [MeSH] |
| #3 Intervention: Guided by ultrasonography | “Ultrasonography” [MeSH] |
| #4 Outcome: Resolution of complications, measured by clinical improvement | “Treatment Outcome” [MeSH] |

**Table S2:** tiab terms

| #1 Population: Patients presenting with vascular complications due to hyaluronic acid dermal filler injections | “vascular complications” [tiab] OR “vascular occlusion“ [tiab] “ischemia” [tiab] OR “skin necrosis” [tiab] OR “filler complications” [tiab] OR “dermal fillers” [tiab] OR “hyaluronic acid” [tiab] |
| --- | --- |
| #2 Intervention: Administration of hyaluronidase | “hyaluronidase” [tiab] OR “hyaluronoglucosaminidase” [tiab] |
| #3 Intervention: Guided by ultrasonography | “sono-guided injection” [tiab] OR “ultrasound-guided injection” [tiab] OR “sonography” [tiab] OR “ultrasound” [tiab] OR “ultrasonography” [tiab] |
| #4 Outcome: Resolution of complications, measured by clinical improvement | “resolution” [tiab] OR “healing” [tiab] OR “treatment outcome” [tiab] |

**Table S3:** Search strategy used for Pubmed/Medline, Scopus, Embase, and Web of Science (Date: 8.24.2024).

| **Search** | **Query** | **Results** |
| --- | --- | --- |
| **Pubmed/Medline:** | | |
| #1 | **"Dermal Fillers" [MeSH] OR "Hyaluronic Acid" [MeSH] OR "Ischemia" [MeSH] OR "Necrosis" [MeSH] OR "vascular complications" [tiab] OR "vascular occlusion "[tiab] "ischemia" [tiab] OR "skin necrosis" [tiab] OR "dermal fillers" [tiab] OR "filler complications" [tiab] OR "hyaluronic acid" [tiab]** | 88,594 |
| #2 | "Hyaluronoglucosaminidase" [MeSH] OR "hyaluronidase" [tiab] OR "hyaluronoglucosaminidase" [tiab] | 12,940 |
| #3 | **"Ultrasonography" [MeSH] OR "sono-guided injection" [tiab] OR "ultrasound-guided injection" [tiab] OR "sonography" [tiab] OR "ultrasound" [tiab] OR "ultrasonography" [tiab]** | 724,537 |
| #4 | **"Treatment Outcome" [MeSH] OR "resolution" [tiab] OR "healing" [tiab] OR "treatment outcome" [tiab]** | 2,033,122 |
| #5 | **#1 AND #2 AND #3 AND #4** | 9 |
| **Scopus:** | | |
| #1 | ( TITLE-ABS-KEY ( "Dermal Fillers" ) OR TITLE-ABS-KEY ( "Hyaluronic Acid" ) OR TITLE-ABS-KEY ( "Ischemia" ) OR TITLE-ABS-KEY ( "Necrosis" ) OR TITLE-ABS-KEY ( "vascular complications" ) OR TITLE-ABS-KEY ( "vascular occlusion" ) OR TITLE-ABS-KEY ( "skin necrosis" ) OR TITLE-ABS-KEY ( "filler complications" ) ) | 1,367,926 |
| #2 | ( TITLE-ABS-KEY ( "Hyaluronoglucosaminidase" ) OR TITLE-ABS-KEY ( "hyaluronidase" ) OR TITLE-ABS-KEY ( "hyaluronoglucosaminidase" ) ) | 19,540 |
| #3 | ( TITLE-ABS-KEY ( "Ultrasonography" ) OR TITLE-ABS-KEY ( "sono-guided injection" ) OR TITLE-ABS-KEY ( "ultrasound-guided injection" ) OR TITLE-ABS-KEY ( "sonography" ) OR TITLE-ABS-KEY ( "ultrasound" ) ) | 823,158 |
| #4 | ( TITLE-ABS-KEY ( "Treatment Outcome" ) OR TITLE-ABS-KEY ( "resolution" ) OR TITLE-ABS-KEY ( "healing" ) ) | 3,887,213 |
| #5 | **#1 AND #2 AND #3 AND #4** | 29 |
| **Embase:** | | |
| #1 | ('injectable dermal implant'/exp OR 'artefill':ti,ab OR 'bellafill':ti,ab OR 'belotero balance':ti,ab OR 'belotero intense':ti,ab OR 'belotero soft':ti,ab OR 'elevess':ti,ab OR 'hydrelle':ti,ab OR 'juvederm hydrate':ti,ab OR 'juvederm ultra plus':ti,ab OR 'juvederm ultra plus xc':ti,ab OR 'juvederm volbella':ti,ab OR 'juvederm volift':ti,ab OR 'juvederm voluma':ti,ab OR 'juvederm voluma xc':ti,ab OR 'revanesse versa':ti,ab OR 'anaesthetic microbe-derived dermal tissue reconstructive material':ti,ab OR 'animal-derived dermal tissue reconstructive material':ti,ab OR 'bioabsorbable synthetic polymer dermal tissue reconstructive material':ti,ab OR 'dermal filler':ti,ab OR 'dermal fillers':ti,ab OR 'dermal injectable filler':ti,ab OR 'dermal tissue reconstructive material':ti,ab OR 'dermal tissue reconstructive material, animal-derived':ti,ab OR 'dermal tissue reconstructive material, microbe-derived':ti,ab OR 'dermal tissue reconstructive material, microbe-derived, anaesthetic':ti,ab OR 'dermal tissue reconstructive material, synthetic mineral':ti,ab OR 'dermal tissue reconstructive material, synthetic polymer, bioabsorbable':ti,ab OR 'dermal tissue reconstructive materials':ti,ab OR 'facial filler':ti,ab OR 'injectable cosmetic filler':ti,ab OR 'injectable cosmetic wrinkle filler':ti,ab OR 'injectable dermal implant':ti,ab OR 'injectable dermal reconstructive implant':ti,ab OR 'injectable facial filler':ti,ab OR 'injectable filler':ti,ab OR 'injectable wrinkle filler':ti,ab OR 'microbe-derived dermal tissue reconstructive material':ti,ab OR 'skin filler':ti,ab OR 'skin fillers':ti,ab OR 'soft tissue filler':ti,ab OR 'soft tissue injectable implant':ti,ab OR 'synthetic mineral dermal tissue reconstructive material':ti,ab OR 'hyaluronic acid'/exp OR 'adant':ti,ab OR 'adant dispo':ti,ab OR 'amo vitrax':ti,ab OR 'amvisc':ti,ab OR 'amvisc plus':ti,ab OR 'arthrease':ti,ab OR 'artz':ti,ab OR 'biolon':ti,ab OR 'bionect':ti,ab OR 'clearvisc':ti,ab OR 'duovisc':ti,ab OR 'durolane':ti,ab OR 'eyecon':ti,ab OR 'go-on (drug)':ti,ab OR 'halonix':ti,ab OR 'healon':ti,ab OR 'healon gv':ti,ab OR 'healon yellow':ti,ab OR 'healon5':ti,ab OR 'healonid':ti,ab OR 'hialid':ti,ab OR 'hyalcon':ti,ab OR 'hyalein':ti,ab OR 'hyalgal':ti,ab OR 'hyalgan':ti,ab OR 'hyalovet':ti,ab OR 'hyalubrix':ti,ab OR 'hyaluronan':ti,ab OR 'hyaluronate':ti,ab OR 'hyaluronate sodium':ti,ab OR 'hyaluronic acid':ti,ab OR 'hyaluronic acid component':ti,ab OR 'hyladerm':ti,ab OR 'hylaform':ti,ab OR 'hylan g f 20':ti,ab OR 'hylan g-f 20':ti,ab OR 'hylartin v':ti,ab OR 'hylo-comod':ti,ab OR 'hylumed':ti,ab OR 'hyruan':ti,ab OR 'ialugen':ti,ab OR 'juvederm':ti,ab OR 'lagricel ofteno':ti,ab OR 'laservis':ti,ab OR 'me 3710':ti,ab OR 'monovisc':ti,ab OR 'na hylan':ti,ab OR 'na-hylan':ti,ab OR 'nrd 101':ti,ab OR 'nrd101':ti,ab OR 'ophthalin':ti,ab OR 'ophthalin plus':ti,ab OR 'orthovisc':ti,ab OR 'ostenil':ti,ab OR 'perlane':ti,ab OR 'potassium hyaluronate':ti,ab OR 'provisc':ti,ab OR 'radiaplexrx':ti,ab OR 'restylane':ti,ab OR 'restylane lyft':ti,ab OR 'si 4402':ti,ab OR 'sinovial':ti,ab OR 'sl 1010':ti,ab OR 'sodium hyaluronate':ti,ab OR 'sperm select':ti,ab OR 'supartz':ti,ab OR 'suplasyn':ti,ab OR 'synocrom':ti,ab OR 'synojoynt':ti,ab OR 'synvisc':ti,ab OR 'teosyal':ti,ab OR 'triluron':ti,ab OR 'unihylon':ti,ab OR 'viscoseal':ti,ab OR 'vismed':ti,ab OR 'vitrax':ti,ab OR 'ischemia'/exp OR 'blood circulation disorder':ti,ab OR 'blood flow disorder':ti,ab OR 'circulation disorder':ti,ab OR 'circulation failure':ti,ab OR 'circulatory disorder':ti,ab OR 'circulatory disturbance':ti,ab OR 'circulatory failure':ti,ab OR 'ischaemia':ti,ab OR 'ischaemic disease':ti,ab OR 'ischaemic episode':ti,ab OR 'ischaemic event':ti,ab OR 'ischaemic syndrome':ti,ab OR 'ischemia':ti,ab OR 'ischemic disease':ti,ab OR 'ischemic episode':ti,ab OR 'ischemic event':ti,ab OR 'ischemic syndrome':ti,ab OR 'tissue ischaemia':ti,ab OR 'tissue ischemia':ti,ab OR 'warm ischaemia':ti,ab OR 'warm ischemia':ti,ab OR 'necrosis'/exp OR 'vascular complications':ti,ab OR 'blood vessel occlusion'/exp OR 'blood vessel obstruction':ti,ab OR 'blood vessel occlusion':ti,ab OR 'obliterative vascular disease':ti,ab OR 'obstructive vascular disease':ti,ab OR 'occlusive vascular disease':ti,ab OR 'vascular obliteration':ti,ab OR 'vascular obstruction':ti,ab OR 'vascular occlusion':ti,ab OR 'vascular occlusive disease':ti,ab OR 'skin necrosis'/exp OR 'cutaneous necrosis':ti,ab OR 'cutaneous necrotic lesion':ti,ab OR 'dermal necrosis':ti,ab OR 'dermatonecrosis':ti,ab OR 'dermo-necrosis':ti,ab OR 'dermonecrosis':ti,ab OR 'embolia cutis':ti,ab OR 'necrosis cutanea':ti,ab OR 'necrosis cutanea sicca':ti,ab OR 'necrosis, skin':ti,ab OR 'necrotic cutaneous lesion':ti,ab OR 'necrotic skin lesion':ti,ab OR 'skin necrosis':ti,ab OR 'skin necrotic lesion':ti,ab OR 'filler complications':ti,ab) AND ('hyaluronoglucosaminidase'/exp OR 'hyaluronidase'/exp) AND ('doppler ultrasonography'/exp OR 'doppler echography':ti,ab OR 'doppler ultrasonography':ti,ab OR 'echography, doppler':ti,ab OR 'ultrasonography, doppler':ti,ab OR 'color doppler flowmetry'/exp OR 'color doppler flowmetry':ti,ab OR 'color doppler':ti,ab OR 'color doppler ultrasonography':ti,ab OR 'color ultrasound flowmetry':ti,ab OR 'doppler color flowmetry':ti,ab OR 'doppler color ultrasound flowmetry':ti,ab OR 'doppler ultrasound color flowmetry':ti,ab OR 'flowmetry, color ultrasound':ti,ab OR 'ultrasonography, doppler, color':ti,ab OR 'ultrasound color flowmetry':ti,ab OR 'interventional ultrasonography'/exp OR 'us-guided intervention':ti,ab OR 'interventional endoscopic ultrasonography':ti,ab OR 'interventional ultrasonography':ti,ab OR 'ultrasonography, interventional':ti,ab OR 'ultrasound-guided intervention':ti,ab OR 'duplex doppler ultrasonography'/exp OR 'duplex doppler ultrasonography':ti,ab OR 'ultrasonography, doppler, duplex':ti,ab OR 'sono-guided injection':ti,ab OR 'ultrasound guided injection'/exp OR sonography:ti,ab OR 'ultrasound'/exp OR 'phonophoresis':ti,ab OR 'radiation, ultrasonic':ti,ab OR 'sonication':ti,ab OR 'sonification':ti,ab OR 'ultra sound':ti,ab OR 'ultrashell':ti,ab OR 'ultrasonic':ti,ab OR 'ultrasonic energy':ti,ab OR 'ultrasonic irradiation':ti,ab OR 'ultrasonic measurement':ti,ab OR 'ultrasonic sound':ti,ab OR 'ultrasonic wave':ti,ab OR 'ultrasonic waves':ti,ab OR 'ultrasonics':ti,ab OR 'ultrasound':ti,ab OR 'ultrasound radiation':ti,ab) AND ('treatment outcome'/exp OR 'health care outcome and process assessment':ti,ab OR 'healthcare outcome and process assessment':ti,ab OR 'medical futility':ti,ab OR 'outcome and process assessment (health care)':ti,ab OR 'outcome and process assessment, health care':ti,ab OR 'outcome management':ti,ab OR 'patient outcome':ti,ab OR 'therapeutic outcome':ti,ab OR 'therapy outcome':ti,ab OR 'treatment outcome':ti,ab OR 'resolution'/exp OR 'healing'/exp OR 'healing':ti,ab) | 24 |
| **Web of Science:** | | |
| #1 | (((((((TS=(( "Dermal Fillers" )) OR TS=("Hyaluronic Acid" )) OR TS=("Ischemia" )) OR TS=("Necrosis" )) OR TS=("vascular complications" )) OR TS=("vascular occlusion")) OR TS=(( "skin necrosis" )) OR TS=("filler complications"))) | 816,710 |
| #2 | ((TS=("Hyaluronoglucosaminidase" )) OR TS=("hyaluronidase" )) OR TS=("hyaluronoglucosaminidase" ) | 8,216 |
| #3 | ((((TS=("Ultrasonography" )) OR TS=("sono-guided injection" )) OR TS=("ultrasound-guided injection" )) OR TS=("sonography" )) OR TS=("ultrasound" ) | 596,717 |
| #4 | ((TS=("Treatment Outcome" )) OR TS=("resolution" )) OR TS=("healing" ) | 1,777,866 |
| #5 | **#1 AND #2 AND #3 AND #4** | 11 |

**Table S4.** Characteristics of the included studies

| **First Author** | **Year** | **Design** | **Sono-guided HAse Injection** | **Follow-up Time** | **Follow-up protocol** | **Cases Gender** | **Cases Age** |
| --- | --- | --- | --- | --- | --- | --- | --- |
| Urso (3) | 2024 | Case Series (5 cases) | Yes | 20 days | Ultrasound | Female | 32.2±3.92 |
| Schelke (2) | 2023 | Case Series (39 cases) | Yes | 1 day | Ultrasound | Female: 36 (92.3%), Male: 3 (7.7%) | 35.3 ±11.8 |
| Schelke (6) | 2023 | Case Series (21 cases) | Yes | NA | Resolved symptoms | NA | ≥18 |
| Munia (7) | 2022 | Case Series (10 cases) | Yes | 5 minutes | Ultrasound | NA | NA |
| Elzen (11) | 2021 | Case Report | Yes | 9 months | Clinical resolution of symptoms | Female | 27 |
| Habib (5) | 2020 | Case Report | Yes | 13 minutes | Ultrasound | NA | 28 |
| Lima (12) | 2019 | Case Report | Yes | 15 minutes | Ultrasound | Female | 29 |
| Schelke (13) | 2018 | Case Report | Yes | 1 day | Clinical resolution of symptoms | Female | 30 |
| Kwon (14) | 2017 | Case Report | Yes | 8 weeks | Ultrasound | Female | 31 |

**Table S5.** Case by case presentation from the included studies

| **Case No** | **HA Site** | **Sign and Symptom** | **Involved Vessels** | **HA-HAse Interval (Days)** | **Grayscale and Color Doppler US Findings** | **Tx Sessions** | **HAse Dosage (IU)** | **Time to Resolve (days)** | **Combination Therapy** | **Outcome** | **First Author** |
| --- | --- | --- | --- | --- | --- | --- | --- | --- | --- | --- | --- |
| 1 | Inferior lip | Lip necrosis | left inferior labial artery | 3 | HA accumulation with impaired arterial flow | 2 | 120-30 | Within 20 days | Oral antibiotic (5 days)/ASA 300 mg (2 weeks) | Healed | Urso (3) |
| 2 | Chin | Skin necrosis | left submental arteries | 1 | HA accumulation with impaired arterial flow | 1 | 75 | Within 20 days | Oral antibiotic (5 days)/ASA 300 mg (2 weeks) | Healed | Urso (3) |
| 3 | NLF | Tissue distress and pustules | nose’s right dorsal artery | 2 | HA accumulation with impaired arterial flow | 1 | 100 | Within 20 days | Oral antibiotic (5 days)/ASA 300 mg (2 weeks) | Healed | Urso (3) |
| 4 | Nose | Tissue distress and pustules | left angular artery branches | 2 | HA accumulation with impaired arterial flow | 2 | 50-30 | Within 20 days | Oral antibiotic (5 days)/ASA 300 mg (2 weeks) | Healed | Urso (3) |
| 5 | Glabella/Nose | Skin scar resulting from necrosis | right dorsal and supratrochlear arteries branches | 38 | HA accumulation with impaired arterial flow | 1 | 30 | Not resolved | Oral antibiotic (5 days) | Stable lesion | Urso (3) |
| 6 | Nose | NA | Angular artery | 1 | NA | 1 | 35 | 0 | ASA, warm pads | Healed | Schelke (6) |
| 7 | Nose | NA | Angular artery | 0.17 | NA | 1 | 65 | 0 | ASA, warm pads | Healed | Schelke (6) |
| 8 | Nose | NA | Angular artery | 1.5 | NA | 2 | 35-35 | 0 | Hyperbaric oxygen, ASA, warm pads | Healed | Schelke (6) |
| 9 | Nose | NA | Superior labial and facial arteries | 1 | NA | 2 | 60-60 | 0 | ASA, warm pads | Healed | Schelke (6) |
| 10 | Lip | NA | Columellar and Superior labial arteries | 0.5 | NA | 1 | 45 | 0 | ASA, warm pads | Healed | Schelke (6) |
| 11 | Lip | NA | Columellar and Superior labial arteries | 0.125 | NA | 1 | 40 | 0 | ASA, warm pads | Healed | Schelke (6) |
| 12 | Lip | NA | Superior labial artery | 0.17 | NA | 1 | 40 | 0 | ASA, warm pads | Healed | Schelke (6) |
| 13 | Lip | NA | Superior labial artery | 3 | NA | 1 | 50 | 0 | ASA, warm pads | Healed | Schelke (6) |
| 14 | Lip | NA | Superior labial artery | 1 | NA | 2 | 40-40 | 0 | Hyperbaric oxygen, ASA, warm pads | Healed | Schelke (6) |
| 15 | Lip | NA | Superior labial artery | 1 | NA | NA | 40 | NA | ASA, warm pads | NA | Schelke (6) |
| 16 | Forehead | NA | Superior labial artery | 0.17 | NA | 1 | 35 | 0 | ASA, warm pads | Healed | Schelke (6) |
| 17 | Forehead | NA | Superior labial artery | 0.33 | NA | 1 | 35 | 0 | ASA, warm pads | Healed | Schelke (6) |
| 18 | Forehead | NA | Superior labial artery | 2.5 | NA | 1 | 150 | 0 | ASA, warm pads | Healed | Schelke (6) |
| 19 | Chin | NA | Submental artery | 4 | NA | 1 | 50 | 0 | ASA, warm pads | Healed | Schelke (6) |
| 20 | Chin | NA | Submental artery | 1 | NA | 1 | 75 | 0 | ASA, warm pads | Healed | Schelke (6) |
| 21 | Chin | NA | Inferior labial artery | 1.5 |  | 1 | 60 | 0 | ASA, warm pads | Healed | Schelke (6) |
| 22 | Chin | Pain, pustular eruptions and tissue necrosis | Inferior labial artery | 56 | NA | 1 | 50 | Not resolved | ASA, warm pads, oral antibiotic | Stable lesion | Schelke (6) |
| 23 | Parietal | NA | Superficial temporal artery | 12 | NA | 2 | 75-50 | 0 | ASA, warm pads | NA | Schelke (6) |
| 24 | Lip | NA | Columellar and Superior labial arteries | 3 | NA | 2 | 45-45 | 0 | ASA, warm pads | Healed | Schelke (6) |
| 25 | Mandibula | NA | Transverse facial artery | 3 | NA | 2 | 80-50 | 0 | ASA, warm pads | Healed | Schelke (6) |
| 26 | Lip | NA | Columellar and Superior labial arteries | 3 | NA | 2 | 40-40 | 0 | ASA, warm pads | Healed | Schelke (6) |
| 27 | Right lateral cheekbone | pain, discoloration, itching, mild crusting, hair loss, and reduced sensation | superior temporal artery | 21 | HA deposit | 1 | 60 | 2 | NA | Healed | Elzen (11) |
| 28 | NA | NA | Superior labial artery | NA | absent or turbulent flow, suggesting vessel blockage | 1 | NA | 0 | NA | Healed | Habib (5) |
| 29 | Cheeks, NLF, chin | Yellowish spot, bilateral peribuccal pallor, and a small, palpable lump on the left side of the lip-chin area | The vessel (not mentioned) showed a narrowed lumen but remained open | 3 | filler accumulationcompromised a blood vessel | 1 | NA | 15 minutes | NA | Healed | Lima (12) |
| 30 | Lower lip | Pain, blanching, crusting | NA | NA | HA deposit | 1 | 150 | 0 | NA | Healed | Schelke (13) |
| 31 | Right NLF | Pain and discoloration the right cheek, livedoid purpuric patches on the right NLF, glabella, and nose, without signs of necrosis | right angular artery | NA | HA pseudocyst  compressing right angular artery | 1 | 1500 | Within 8 weeks | Sublingual nitroglycerin and ASA 100 mg (1week) | Healed | Kwon (14) |
| 32-70 (39 cases reported in an accumulative way) | Chin: 41.03%, Upper lip: 43.59%, Nose: 38.46%, Nasolabial fold: 25.64%, Temple: 5.13%, Lower lip: 17.95%, Lateral cheek: 7.69%, Corner of the mouth: 7.69%, Midface: 25.64%, Forehead: 5.13%, Jawline: 7.69%. | Erythema: 71.79%, Blanching: 28.21%, Edema: 28.21%, Livido reticularis: 41.03%, Pustula: 7.69%, Pain: 43.59%, Itching: 2.56%, Hematoma: 2.56%. | NA | 0 in 97.4% and a couple of hours in 2.6% | Decreased blood flow with minimal to absent vascular signals during Doppler ultrasound | 1 session in 84.6% and 2 sessions in 15.4% with 24 hours interval | 95±65 (first dose), 51±10 (second dose) | 0 (except for hematoma) | Fucidin 20 mg/g ointment, intense pulse light for erythema following skin necrosis | Healed (100%) | Schelke (2) |
| 70-80 (10 patients reported in an accumulative way) | Various parts of face | Livedo reticularis: 100%, hypoesthesia: 50%, local pain: 20% | NA | 0.33 (0.125-3) | No visible blood flow and tardus parvus pattern on initial Doppler ultrasound evaluation | Repeated as needed and stopped once ultrasound confirmed adequate flow | 500 (300-750) | 0 | No topical treatments; two with nasal ala ischemia received IV vasodilator (Alprostadil for 72 hours) | Healed (100%) | Munia (7) |

**Footnote:** HA: Hyaluronic Acid; Hase: Hyaluronidase; US: Ultrasound; ASA: Acetylsalicylic Acid; Tx: Treatment; NLF: Nasolabial Fold; IU, International Units; NA, Not Applicable.
